# Integrating CBAM and Squeeze‐and‐Excitation Networks for Accurate Grapevine Leaf Disease Diagnosis

**DOI:** 10.1002/fsn3.70377

**Published:** 2025-06-02

**Authors:** Yavuz Unal

**Affiliations:** ^1^ Engineering and Architecture Faculty, Computer Engineering Department Sinop University Sinop Turkey

**Keywords:** CBAM, deep learning, grapevine leaf disease, SENet

## Abstract

The vine plant holds significant importance beyond grape farming due to its diverse products. Various grape‐derived products, such as wine and molasses, highlight the vine plant's role as a valuable agricultural resource. Additionally, traditional cuisines around the world widely utilize grape leaves, contributing to their substantial economic value. However, diseases affecting grape leaves not only harm the plant and its yield but also render the leaves unsuitable for culinary use, leading to considerable economic losses for producers. Detecting diseases on grape leaves is a challenging and time‐consuming task when performed manually. Thus, developing a deep learning‐based model to automate the classification of grape leaf diseases is of critical importance. This study aims to classify the most common grape leaf diseases grape—scab (grape leaf blister mite) and downy mildew (grapevine downy mildew) alongside healthy leaves using deep learning techniques. Initially, we conducted a basic classification using pre‐trained deep learning models. Subsequently, the Convolutional Block Attention Module (CBAM) and Squeeze‐and‐Excitation Networks (SE) were integrated into the most successful pre‐trained classification model to enhance classification performance. As a result, the classification accuracy improved from 92.73% to 96.36%.

## Graphical abstract

This study presents a deep learning‐based model for classifying grape leaf diseases, including grape scab and downy mildew. Initially, pretrained models were used, followed by the integration of CBAM and SE attention modules to improve performance. The proposed approach increased classification accuracy from 92.73% to 96.36%, highlighting its effectiveness for automated grapevine disease diagnosis.

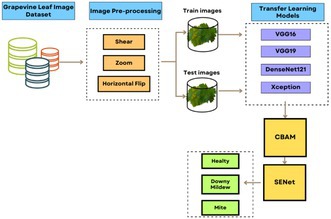

## 1 Introduction

Agriculture plays a vital role in meeting the basic nutritional needs of humans and animals. Today, due to various factors such as the increasing human population and limited resources, agriculture has become one of the areas of strategic importance. The integration of new technologies into the agricultural sector allows agricultural activities to be carried out more efficiently and easily (Kunduracioglu and Pacal 2024).

The grapevine plant ( *Vitis vinifera* ) has a wide distribution area throughout the world and has important economic value. Grapes are one of the most consumed fruits in the world, and are consumed as table fruit, as well as being used in various industrial and commercial areas such as wine grapes, dried grapes, must, molasses, vinegar, grape seeds, and seed oil, and grape juice production (Bouby et al. 2023).

Additionally, traditional cuisines in some parts of the world strongly favor vine leaves, a use that has created a multi‐million dollar market with significant economic value (Koklu et al. 2022).

If not identified early and promptly, diseases that affect grapevine leaves can result in significant economic losses (Huang et al. 2020).

Detection of plant diseases by traditional methods is usually based on visual inspection and expert opinion. However, these methods can lead to high costs and time loss, as well as increased risk of error. This is particularly true in large vineyard areas, where accurate and rapid diagnosis of diseases becomes significantly challenging (Xie et al. 2020).

To meet the food needs of the increasing population all over the world, the implementation of technology‐based production methods in large agricultural lands has become mandatory. In this direction, the use of deep learning, machine learning, and image processing techniques has become rapidly widespread in the agricultural sector in recent years. These technologies play a critical role in the early and accurate detection of agricultural diseases (Lukic et al. 2017).

Early and accurate detection of grapevine leaf diseases is crucial for farmers. Automating the classification process through computer systems will reduce the workload of those who manually detect these diseases and significantly speed up the process (Manavalan 2020). Convolutional Neural Network (CNN) architectures have been extensively employed in agricultural applications based on image analysis in recent years, particularly in the identification of leaf diseases (Patrício and Rieder 2018).

The primary aim of this work is to employ deep‐learning techniques to identify grapevine leaf diseases. Our objective is to employ sophisticated deep‐learning methodologies to create a system capable of precisely identifying diverse grapevine leaf diseases. This study seeks to enhance agricultural technology by offering an efficient instrument for the early diagnosis and classification of illnesses affecting grapevine leaves utilized in traditional cuisines.

Diseases frequently encountered in vineyards negatively affect plant health and yield, leading to significant economic losses. One of these diseases is the vineyard mite, also known as the grape leaf blister mite.

This disease, caused by a mite known as *Colomerus vitis* (formerly known as *Eriophyes vitis*) that cannot be seen with the naked eye, is quite common in vineyards and causes serious damage to the plant. This condition, also known as “vineyard mite” among the public, is referred to by different names and is also referred to as “mites” in the literature (Dennill 1991). Mites settle on the leaves, buds, and branches of the vine plant during the winter months and feed on plant sap. The absorption of plant sap from the lower surfaces of the leaves causes silvery and mold‐like structures to form in these areas. On the upper surfaces of the leaves, the absorption of plant sap results in the formation of deformities and pimple‐like structures. In the later stages of the disease, deformations in the leaves cause the plant to be undernourished and to suffer from developmental losses (Guedes et al. 2024).

Grapevine downy mildew, in addition to grapevine mite disease, is a disease that frequently occurs in vineyards and causes significant damage.

Plasmopara viticola is the fungus that causes the illness. It infects the vine plant's leaves, branches, and fruit clusters. It spreads quickly and is most common during times of intense rainfall. Early on in the disease's progression, the leaves develop yellowish patches that resemble oil stains (Gobbin et al. 2005). Later on, these spots result in dry patches on the leaf, which prevents the leaf from performing its intended role. Additionally, this disease affects the clusters of grapes as well as the leaves, which results in the grapes falling (Koledenkova et al. 2022).

In this study, we aim to integrate the Convolutional Block Attention Module (CBAM) and Squeeze‐and‐Excitation Networks (SE) modules to increase the classification accuracy and the basic classification process that uses pre‐trained neural networks to achieve high classification accuracy. To enhance vineyard management applications and improve the precision and effectiveness of grapevine leaf categorization, we employ deep learning algorithms. In conclusion, this work can help the agriculture industry since our goal is to leverage deep learning to improve the capacity of grapevine leaf disease systems.

## 2 Related Works

In recent years, many studies have been conducted on the classification of grape leaf diseases. These studies provide comprehensive analyses of the detection of vine leaf diseases using different datasets and methodologies. In this context, summary information on relevant studies in the literature is presented below. In order to accurately and quickly diagnose six different diseases (anthracnose, brown spot, mites, black rot, downy mildew, and leaf scorch) observed on grape leaves, Liu et al. (2020) developed an improved deep learning model. In this study, they extracted multidimensional features using the Inception structure and performed classification with a CNN‐based model they named DICNN. The model achieved a classification accuracy of 97.22%. Hernández et al. (2024) conducted an image‐processing‐based study for the early diagnosis of downy mildew, an important disease in viticulture. In this study, they used CNN and Grad‐CAM methods to classify healthy and infected leaves of the Tempranillo grape variety. In the detection of downy mildew, a classification accuracy of 99% was achieved, and a 10‐fold cross‐validation method was applied in the evaluation of the model. Kunduracioglu and Pacal (2024) used two separate grape leaf datasets in their study. The first dataset is the PlantVillage dataset, in which they classified three different diseases observed on grape leaves along with healthy leaves. The second dataset consists of 500 images and includes five different grape varieties. In their studies, they tested 14 different CNN and 17 different vision transformer models on these two datasets and achieved 100% classification accuracy. Patil and More (2024) extracted features using pre‐trained ResNet50 and ResNet101 networks to accurately and quickly detect grape leaf diseases, combined these features, and performed classification with SVM. In the study, a high F1‐score value of 99.82% was achieved. Yadav et al. (2024) classified a dataset consisting of grape leaf images from four classes using VGG16, VGG19, and ResNet50. They achieved a 95% classification success with the ResNet50 model. Karthik et al. (2024) developed a model in their study that includes two different networks, Swin Transformer and Group Shuffle Residual DeformNet (GSRDN). This model was tested on the PlantVillage dataset and achieved an accuracy rate of 98.6%. Muneshwar et al. ( 2024), in their study, achieved 91% test accuracy in distinguishing between diseased and healthy grape leaves using a convolutional neural network (CNN) model. Another study aimed at classifying grape leaf diseases was conducted. In their study, they used DCNN and VGG16 models on both augmented and non‐augmented datasets. As a result of this study, the training and test accuracy rates were obtained as 99.18% and 99.06%, respectively. Huang et al. (2020) applied various deep learning models on a dataset consisting of grape leaf images, which includes four diseased and one healthy class, making a total of five classes. Among these models are Vanilla CNN, VGG16, improved VGG16, MobileNet, improved MobileNet, AlexNet, improved AlexNet, and Ensemble methods. They achieved 100% classification accuracy with the ensemble method. Studies in this area are given in Table 1.

**TABLE 1:** Overview of recent studies on grapevine leaf disease classification.

Authors	Method(s)	Model architecture	Dataset	Number of classes/disease	Accuracy
Liu et al. 2020	Feature extraction + Classification	InceptionV3 + Custom CNN (DICNN)	Custom dataset	6 diseases (Anthracnose, Brown Spot, Mites, Black Rot, Downy Mildew, Leaf Scorch)	97.22%
Hernández et al. 2024	Deep learning	CNN+ Grad‐CAM	Custom (Tempranillo grape leaf)	2 classes/(Healthy, Downy mildew)	99%
Kunduracioglu and Pacal 2024	Deep Learning	14 CNN + 17 vision transformers	PlantVillage & Custom dataset	3 diseases +1 healthy	100%
Patil and More 2024	Feature extraction + Machine Learning	Resnet50 ResNet101+ SVM	Kaggle	3 diseases +1 healthy	99.70%
Yadav et al. 2024	Transfer learning	VGG16 VGG19 ResNet50	Custom dataset	3 diseases +1 healthy	95%
Karthik et al. 2024	Hybrid model	Swin Transformer + GSRDN	PlantVillage	3 diseases +1 healthy	98.6%
Muneshwar et al. 2024	CNN based	CNN	PlantVillage	3 diseases +1 healthy	91%
Prasad et al. 2024	Deep Learning	DCNN+VGG16	Kaggle	3 diseases +1 healthy	100%
Huang et al. 2020	Transfer learning + Data Augmentation	Vanilla CNN VGG16 MobileNet Improved MobileNet AlexNet Improved AlexNet Ensemble	PlantVillage	4 diseases +1 healthy	100%

## 3 Material and Methods

In this study, a deep learning‐based approach has been developed to accurately diagnose diseases in grape leaves. The proposed model combines the CBAM (Convolutional Block Attention Module) and Squeeze‐and‐Excitation Networks (SE‐Net) structures that use attention mechanisms, enabling the model to learn spatial and channel features more effectively. In this section, the dataset used, preprocessing steps, architecture of deep learning models, and training processes are discussed in detail.

The dataset used in the study includes Downy mildew and Grape leaf blister mite diseases, which are frequently observed on vine leaves and cause significant losses in agricultural production and healthy vine leaves. These diseases manifest visually on the leaves with distinct symptoms such as spots and deformities. The proposed model is designed to enhance the performance of pre‐trained networks that classify these visual variations.

### 3.1 Dataset

**FIGURE 1:**
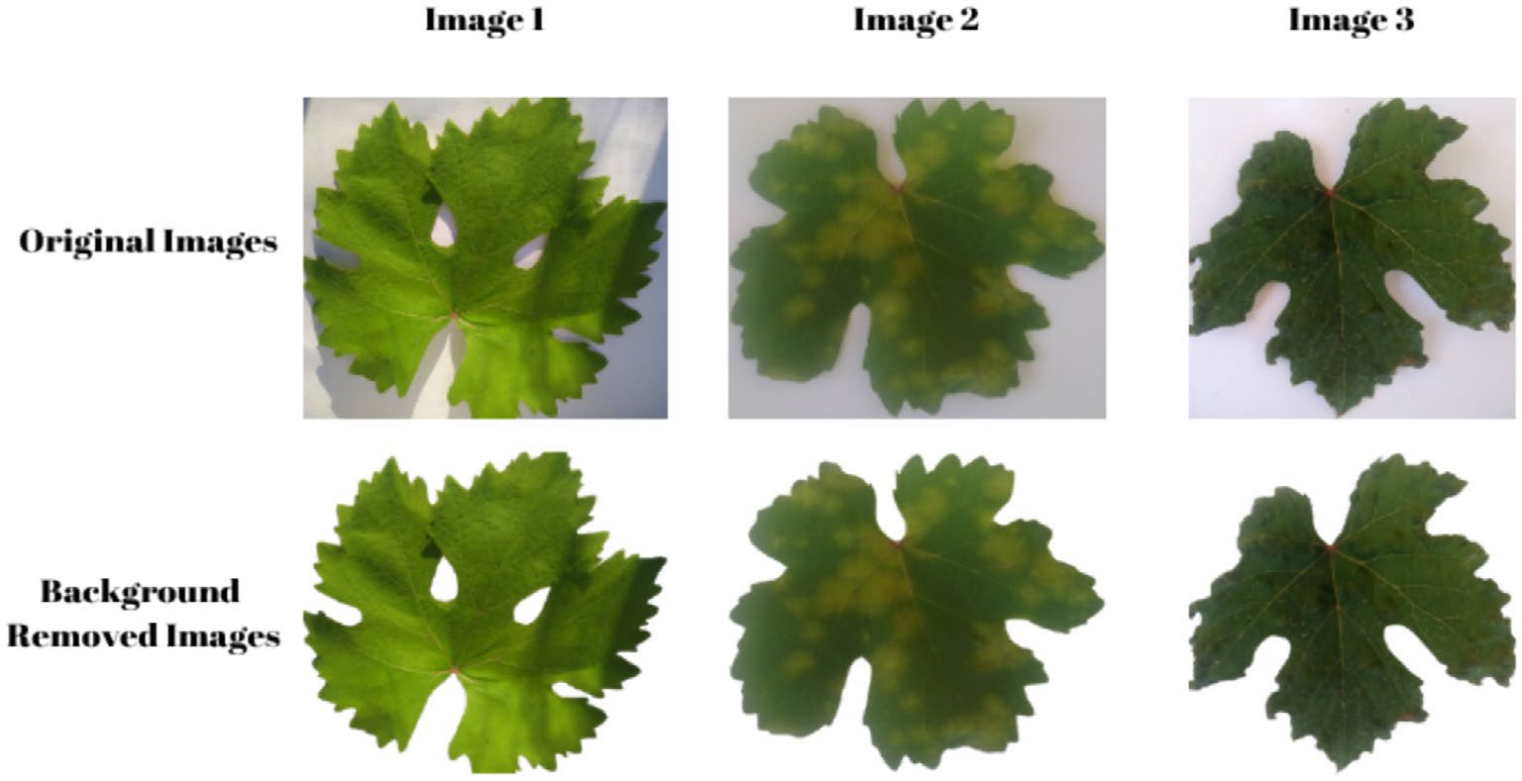
Raw and pre‐processed sample images.

**FIGURE 2:**
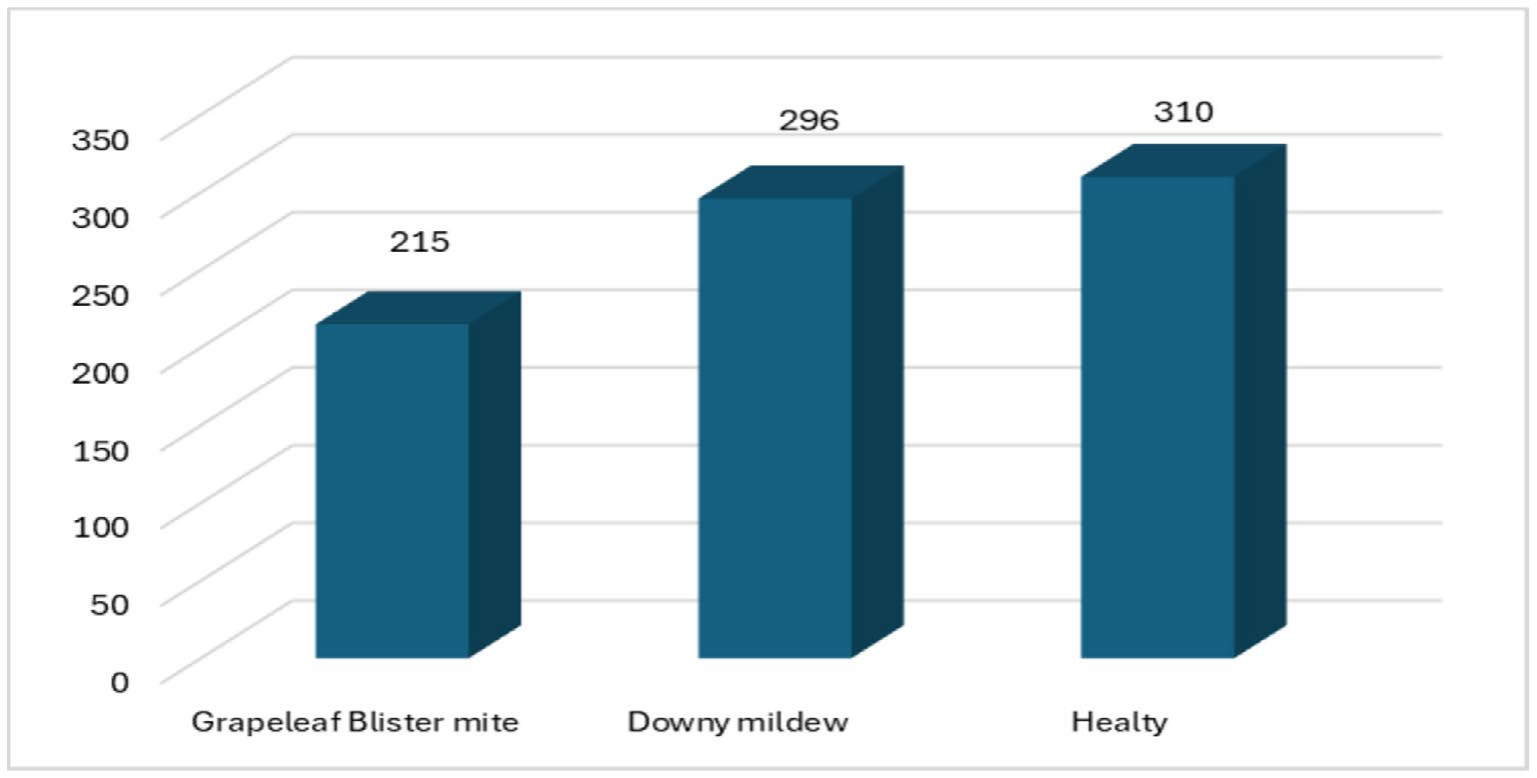
Distribution of classes in the dataset.

The images used in the study were collected specifically for this research. The images were obtained from vineyards in Tokat province of Turkey and were obtained from a vineyard where the Narince vine leaf species specific to the region was found. The original images were saved in RGB color space, 2056 × 1024‐pixel resolution, and JPG file format. The images were taken with a mobile phone with a 13‐megapixel resolution. These images are divided into three main categories, and there are 821 in total. Data collection took place during the growing season between May and June 2023. Images were captured in natural light conditions. Average file size is 3.2 MB. The original images in the dataset include a background; however, this background was removed during the preprocessing stage. Examples of the original images with the background and the pre‐processed images are presented in Figure 1. In addition, the number of images belonging to each class is given in detail in Figure 2.

In the dataset, the number of healthy grapevine leaves is 310, the number of grapevine leaves affected by grapevine downy mildew disease is 296, and the number of grapevine leaves affected by grape leaf blister mite is 215.

### 3.2 Image Pre‐Processing

Several pre‐processing steps were applied to remove the blurring in the original images and to remove unnecessary elements from the background. In this process, firstly, contrast enhancement was performed to solve the blurring problem; then the backgrounds of the images were removed, and finally re‐saved in PNG format with a size of 484 × 515 pixels. The original images and the sample images with the background removed and resized are presented in Figure 1.

### 3.3 Augmentation

In this study, ImageDataGenerator is used for data augmentation operations. Instead of creating new data samples, data augmentation allows the images in the existing dataset to be modified with various transformations to create more diversity. In this context, each transformation (e.g., shear, zoom, flip, etc.) creates different variations of the original images and does not increase the number of images in the dataset. This method allows the model to learn and generalize better by “seeing” different variations of the same image.

The images used in the study were normalized by dividing the pixel values by 255. This normalization process was performed to provide a more stable learning process. Within the scope of data augmentation processes, a 20% shear (shift) was applied to the images, which created a perspective change by extending one side of the image and shortening the other. In addition, a 20% zoom‐in and zoom‐out process was applied to the input images, allowing certain parts of the images to be analyzed in more detail. Finally, a horizontal flip process was performed, which made it easier for the model to recognize images from different angles, increasing generalization performance.

### 3.4 Proposed Model

This section describes the proposed method for the classification of two different diseases observed on grapevine leaves and healthy leaf samples.

**FIGURE 3:**
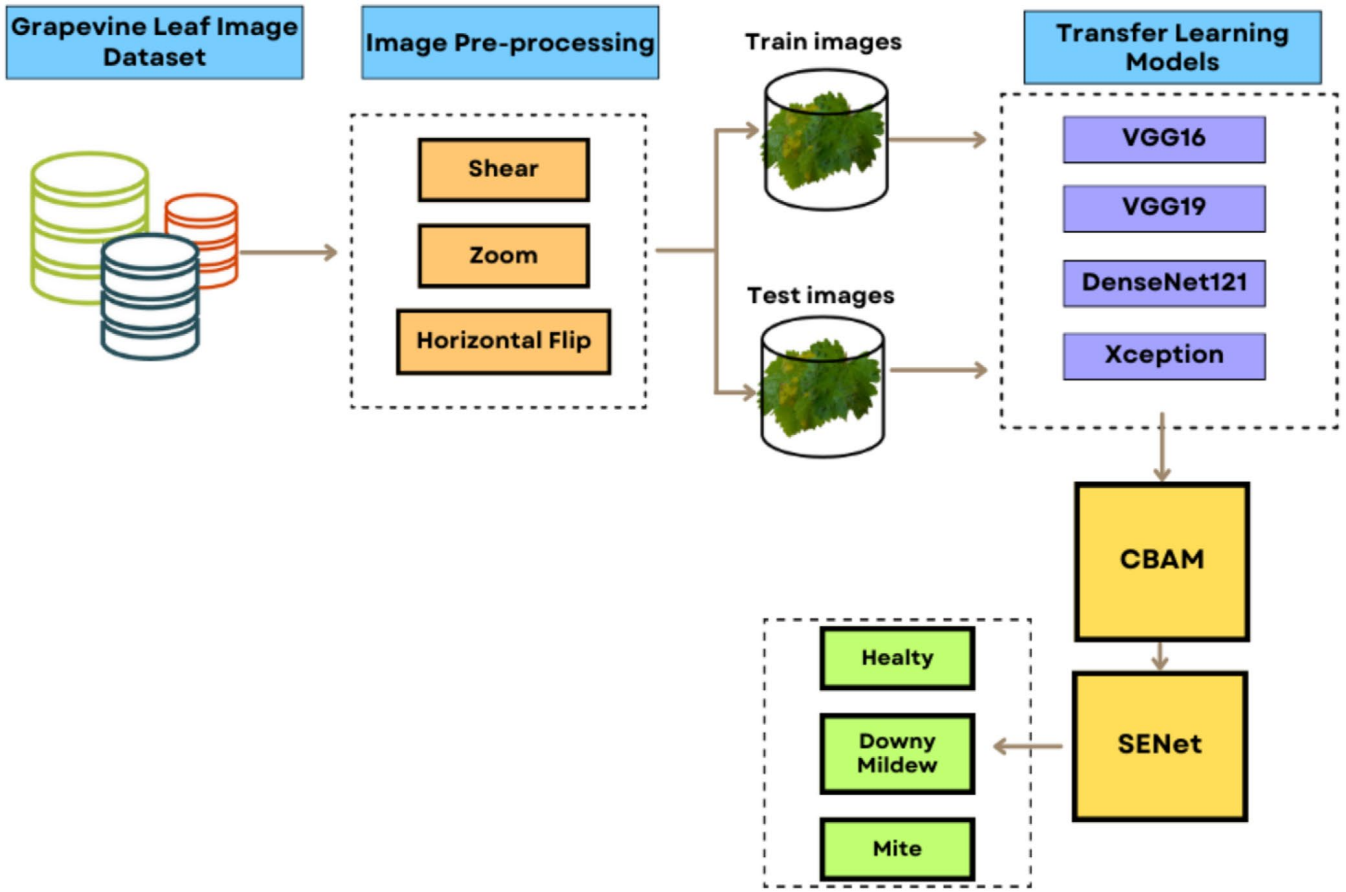
Workflow of our model architecture.

In this study, we developed an innovative hybrid model integrating Convolutional Block Attention Module (CBAM) and Squeeze‐and‐Excitation Networks for grapevine leaf diseases classification. The proposed deep learning‐based model offers significant advantages over traditional manual diagnostic methods. First, it provides significant benefits in terms of time efficiency and cost effectiveness. Unlike manual disease identification, which requires skilled pathologists to visually inspect each leaf individually, our automated approach can rapidly analyze hundreds of leaf images and reduce diagnostic time from hours to seconds per sample. In addition, it will help in early diagnosis and provide convenience for those who do not know about the diseases. The general structure of the proposed approach is given in Figure 3. As a first step, pre‐processing steps such as contrast correction, background removal, and resizing were applied to the images. These processes aim to increase the quality of feature extraction. Pre‐processing also has the potential to affect the performance of the model. After pre‐processing, the data set was divided into training and test data. This ratio was determined as 80% training and 20% test.

Grape leaf images that were preprocessed and separated into training and test were classified with pre‐trained neural network models VGG16, VGG19, DenseNet121, Xception, and InceptionV3. As a result of the analyses, the DenseNet121 model showed the best classification performance, and subsequent analyses were carried out on this model. The main purpose of choosing the DenseNet121 model is to maximize the classification accuracy. In this process, SENet was applied to the DenseNet121 model, and the CBAM mechanism was integrated. Finally, a strengthened structure was created by combining the DenseNet121 model with SENet and CBAM. The experimental results obtained are presented in Table 4.

In this section, the theoretical background of the methods used in the study is briefly explained.

### 3.5 Deep Learning Approach

Deep learning, inspired by the principles of the human brain, is a subfield of machine learning (ML) and artificial intelligence (AI). Its capacity to identify intricate patterns and extract valuable information from massive data sets attracts attention. This technology learns intricate relationships in data and applies them to provide precise classifications or predictions using multi‐layered artificial neural networks. Deep learning networks can represent increasingly complicated data by learning more abstract representations at each layer.

Deep learning typically uses artificial neural networks with three primary components: the input, hidden, and output layers. After the input layer receives data, the output layer performs the final classification or prediction, and the hidden layers learn increasingly complex features. By learning increasingly intricate and abstract properties at each layer, these networks uncover deep structures in the data (Taye 2023).

Transfer learning attempts to apply the classification ability of a model trained on a large number of images to other datasets.

### 3.6 Pre‐Trained Networks

#### 3.6.1 DenseNet121

It is one of the pre‐trained deep learning models that works well for various tasks, such as segmenting and classifying images. This model is a DenseNet architectural variation. The number 121 in its name refers to the fact that it has 121 levels. Because of this characteristic, researchers use it a lot. “Dense connections” are used in this architecture to send the layers' output as input to other layers. As a result, the network can learn to the deep layers more easily. Furthermore, there are some benefits to this model's limited number of parameters. For example, minimal hardware is used to achieve excellent performance (Petchiammal and Murugan 2025).

#### 3.6.2 VGG16

Often utilized for image processing applications, VGG16 is a convolutional neural network (CNN) model with a multi‐layer deep learning architecture. The visual geometry group (VGG) created VGG16, a simple 16‐layer structure. This methodology has demonstrated particular efficacy in classification‐related tasks (Simonyan and Zisserman 2014).

#### 3.6.3 VGG19

It is an improved version of the VGG16 model. It has been improved by adding more layers. It has 19 layers. It uses 3 × 3 convolutional filters. It contains 16 convolutional and 3 fully connected layers. It has maximum summation layers in addition to the ReLU activation function. It accepts VGG19 inputs of size 224 × 224 × 3. It contains about 143 million parameters and requires additional memory (Meena et al. 2022).

#### 3.6.4 InceptionV3

This pre‐trained neural network architecture was developed by Google. Although it can be used in many areas such as object detection, it is especially widely used in the field of image classification. It is a different version of the Inception architecture and was introduced in 2015. One of the pre‐trained models, InceptionV3, can be used within different datasets. It tends to have low overfitting (Szegedy et al. 2017).

#### 3.6.5 Xception

The name Xception was derived from the name Extreme Inception. Created by Google, it is similar to InceptionV3. For more efficient feature extraction, it uses depthwise separable convolutions. To lower computational costs, it divides traditional convolutions into two steps. It is designed to work well with massive datasets. Object detection, image classification, and other computer vision tasks are all done with it (Chollet 2016).

### 3.7 Convolutional Block Attention Module (CBAM)

Attention mechanisms are a crucial technique that enhances the power and efficiency of deep learning networks. These strategies enhance the learning process by enabling the model to concentrate on particular regions or features. The Convolutional Block Attention Module (CBAM) exemplifies these systems, comprising two primary components: the Channel Attention Module and the Spatial Attention Module (Suo et al. 2024).

The Channel Attention Module produces summary data for each channel and utilizes this data to create channel‐level attention coefficients. During this procedure, each channel of the input feature map is examined individually. Initially, summary information for each channel is acquired by global average pooling and global maximum pooling processes. These processes yield statistical summaries that assist in assessing the significance of the channels. Subsequently, this summary information is amalgamated via fully connected layers, resulting in the generation of an attention coefficient for each channel. These coefficients assist the model in identifying the significance of various channels, allowing it to concentrate on more pertinent areas of the data (Woo et al. 2018).

**FIGURE 4:**
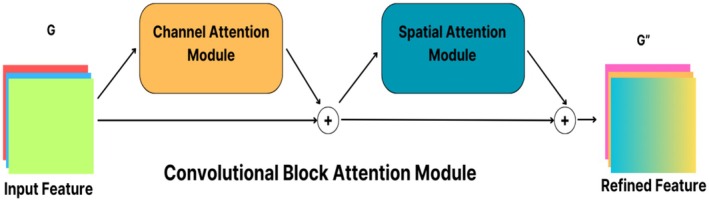
Structure of Convolutional Block Attention Module.

Furthermore, the Spatial Attention Module employs the attention mechanism at the spatial dimension. This module assesses the significance of particular regions within the feature map. The Channel Attention Module and the Spatial Attention Module collaborate, enabling CBAM to execute efficient attention processes at both the channel and spatial dimensions. This combination aids the model in optimizing feature selection and enhancing processing efficiency (Ma et al. 2024) (Figure 4).

### 3.8 Squeeze‐and‐Excitation Networks

SENet (Squeeze‐and‐Excitation Network), a crucial architectural component in Convolutional Neural Networks (CNNs), markedly enhances model performance by emphasizing channel interdependencies. The fundamental element of SENet, the “Squeeze‐and‐Excitation” (SE) block, possesses the capability to adaptively adjust feature responses on a channel‐specific basis, particularly through the modeling of channel dependencies. This approach enhances the significance of the information processed in each layer of a CNN and enables the model to concentrate on more critical areas of the data.

The SE block comprises two primary stages: Squeeze and Excitation. During the compression phase, the spatial dimensions of each channel are reduced through the application of global average pooling. This approach generates a singular scalar value for each channel, encapsulating the overarching significance of the channel. This condensed information enhances the understanding of channel‐level dependencies. During the stimulation phase, the summary data acquired in the compression phase is processed via completely connected layers and activation functions. This approach generates an attention weight for each channel. These coefficients enable the model to discern the significance of various channels and to adjust the responses of the pertinent channels accordingly.

**FIGURE 5:**
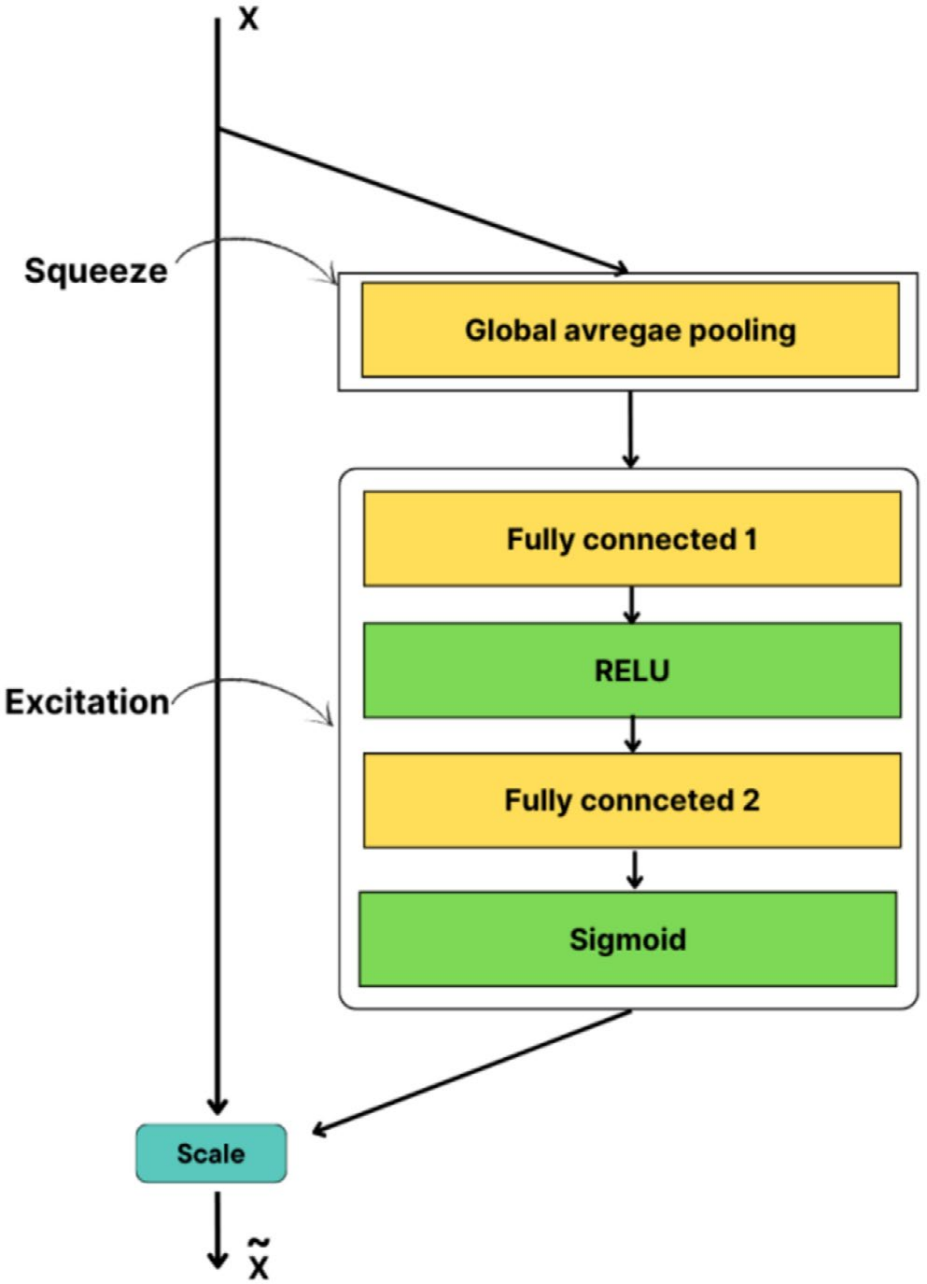
Structure of the Squeeze‐and‐Excitation Block.

The attention mechanism of SENet facilitates channel‐level information processing alongside the convolution operator present in conventional CNNs. Traditional convolution processes primarily emphasize the extraction of spatial data, but the SE block of SENet integrates both spatial and channel‐level information. This enables the model to acquire more informative and distinguishing traits. Specifically, emphasizing channel interactions enhances the model's generalization capability and yields superior performance (Hu et al. 2020) (Figure 5).

### 3.9 Experimental Evaluation Metrics

The model was trained for 50 epochs. The confusion matrix was used to evaluate the validation performance of the model. This matrix shows the correct and incorrect predictions of the classification model in detail and is an important tool for analyzing the success of the model. In the evaluation made through the confusion matrix, the following four basic terms are used to determine the accuracy of the model:

TP: when the predicted results are correctly classified as positive.

TN: when the model correctly classifies the negative class as negative.

FP: when the model incorrectly classifies the negative class as positive.

FN: when the model incorrectly classifies the positive class as negative.

Using these four basic terms (TP, TN, FP, FN), various performance metrics of the model can be calculated:

The percentage of true positive or true negative results in an accurate testing set is known as accuracy. This equation is used to calculate it:

(1) $$\text{Accuracy}=\left(\mathrm{TP}+\mathrm{TN}\right)/\left(\mathrm{TP}+\mathrm{FP}+\mathrm{FN}+\mathrm{TN}\right)$$

where the parameters exist, the metrics employed to assess specificity, sensitivity, and accuracy in performance evaluation encompass TP (True Positives), TN (True Negatives), FP (False Positives), and FN (False Negatives). The quantity of accurately anticipated positive classes is represented by TP. The result of correctly predicted negative classes is TN. The quantity of cases anticipated to be positive yet ultimately classified as negative is referred to as false positives (FP). The quantity of cases anticipated to be negative when the results should have been positive is now denoted as FN.

Precision is the ratio of correctly predicted positive instances to the total number of instances projected as positive, reflecting the accuracy of positive forecasts. The subsequent formula for calculation is as follows:

(2) $$\text{Precision}=\mathrm{TP}/\left(\mathrm{TP}+\mathrm{FP}\right)$$

The ratio of correctly predicted positive instances to the total number of actual positive instances is referred to as recall (sensitivity). This equation is employed for its calculation:

(3) $$\text{Recall}=\mathrm{TP}/\left(\mathrm{TP}+\mathrm{FN}\right)$$

In classification problems, the f‐score is a statistic that evaluates the precision of positive predictions against the recall of successfully identified positive classes. The subsequent formula is the calculation (Sofuoğlu and Birant 2023):

(4) $$F\text{score}=2\times \left(\text{Precision}\times \text{Recall}\right)/\text{Precision}+\text{Recall}$$

## 4 Experimental Results and Discussion

### 4.1 Experimental Setting

**FIGURE 6:**
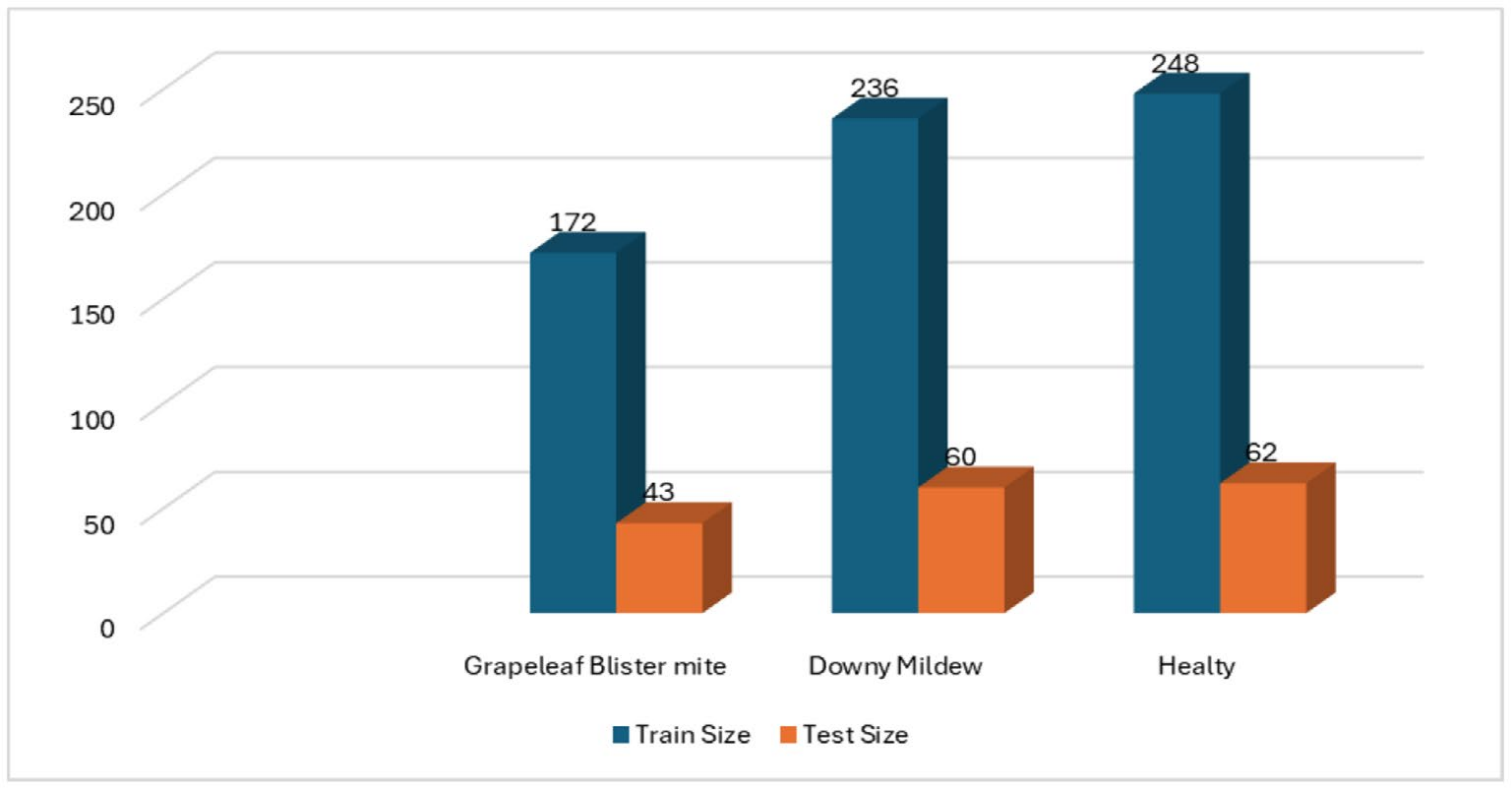
Class distribution of training and test data.

The research was conducted in a cloud environment with an NVIDIA Turing T4 GPU with 16 GB of memory, utilizing Python programming. The resolution of the input images was adjusted based on the architecture of the pre‐trained neural networks employed: 224 × 224 for VGG16 and VGG19, 299 × 299 for InceptionV3 and Xception, and 224 × 224 for DenseNet121. The Adam optimizer was used during training, with a batch size of 32 for both training and testing. The maximum number of training epochs was set to 50, with early stopping implemented to prevent overfitting. A learning rate of 0.0001 was used throughout the training process. The dataset was partitioned into training and testing sets in an 80:20 ratio. The distribution of training and testing samples for each class is illustrated in Figure 6. The primary evaluation metrics utilized to assess the model's performance included accuracy (Acc), precision (Prec), recall (Rec), and F1‐score.

Several parameters were used to establish the experiment as described in Table 2.

**TABLE 2:** Model training hyperparameters.

Parameters	Value
Learning rate	0.0001
Optimizer	Adam
Batch size	32
Epoch	50
Activation function	ReLu

In this study, the learning rate was set to 0.0001 during model training, and the Adam Optimization Algorithm was utilized for the optimization process. The batch size for each mini‐batch was configured to 32, and the ReLU activation function was employed. The training process was conducted over 50 epochs.

### 4.2 Result and Discussion

The proposed deep learning model increased the classification accuracy of the existing DenseNet121 model from approximately 92% to 96.36% in the detection of grapevine downy mildew, grape leaf blister mites, and healthy grapevine leaves, and provided a promising performance increase in this direction. The results obtained in this section are discussed in detail.

The classification performances obtained as a result of the analyses are presented comparatively in Table 3.

**TABLE 3:** Comparison of classification performance (%) different models.

Model	Val‐accuracy	Train accuracy	Precision	Recall	F1‐score
VGG19	84.85%	92.38%	0.86	0.84	0.84
VGG16	85.45%	96.49%	0.86	0.86	0.85
InceptionV3	91.52%	96.04%	0.93	0.91	0.92
Xception	92.12%	94.66%	0.92	0.92	0.92
DenseNet121	92.73%	96.16%	0.93	0.93	0.93
DenseNet121 + SENet	94.55%	96.95%	0.94	0.94	0.94
DenseNet121 + CBAM	95.15%	97.56%	0.95	0.94	0.95
DenseNet121 + CBAM + SENet	96.36%	97.87%	0.96	0.96	0.96

When the accuracy values obtained in the training and validation phases are examined in Table 3, significant performance differences are observed between the models. Traditional VGG architectures (VGG19 and VGG16) showed the lowest success with 84.85% and 85.45% validation accuracy, respectively. More advanced architectures InceptionV3 and Xception significantly increased their performance by reaching 91.52% and 92.12% validation accuracy, respectively. The performance of DenseNet121‐based models is remarkably high in both the basic DenseNet121 and its enhanced versions. In particular, the integration of SE blocks (SENet) and attention mechanisms (CBAM) further increased the validation accuracy of the model. The DenseNet121 + CBAM + SENet model achieved superior success in grapevine leaf disease diagnosis by exhibiting the best performance with 96.36% validation accuracy and 97.87% training accuracy.

Precision, recall, and F1‐score metrics of VGG19 and VGG16 (around 0.84–0.85) are significantly lower than DenseNet121 variants. The same metrics are higher than VGG16 and VGG19 for InceptionV3 and Xception. The DenseNet121 + CBAM + SENet model showed the best performance (0.96) in all three metrics. This shows that the classification performance of the model is balanced and can distinguish both positive and negative classes well.

**FIGURE 7:**
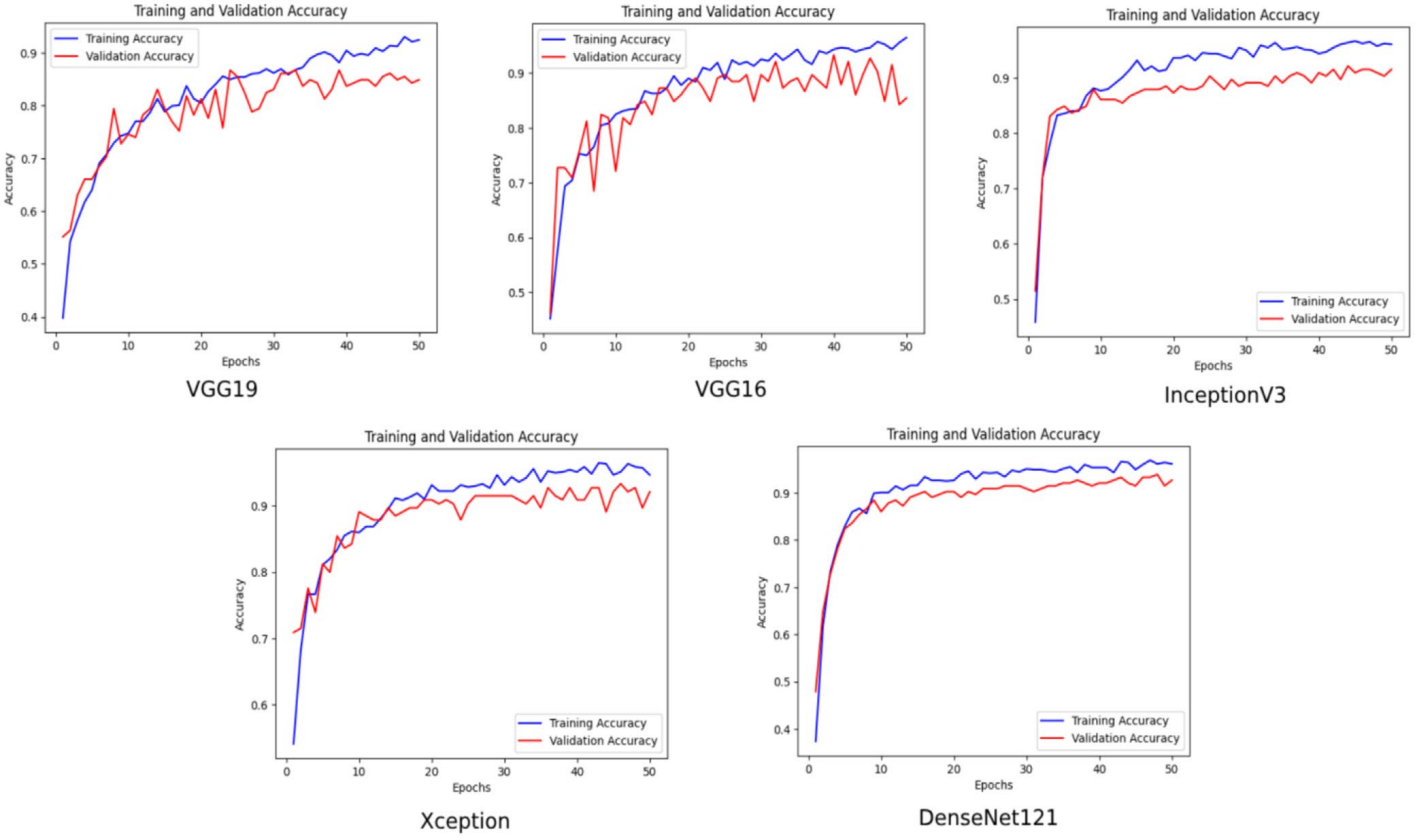
Comparison of training and validation accuracy graphs of pre‐trained networks.

The changes in training and validation accuracy values over time for different deep learning models (VGG19, VGG16, InceptionV3, Xception, and DenseNet121) are presented in Figure 7.

It was observed that the training and validation accuracy rates in all models showed a steady increase from the initial low levels. This situation reveals that the models were able to learn the dataset during the training process. DenseNet121 and Xception models showed a more stable and higher performance in validation accuracy compared to other models. These models seem to be superior to the others in terms of generalization ability.

**FIGURE 8:**
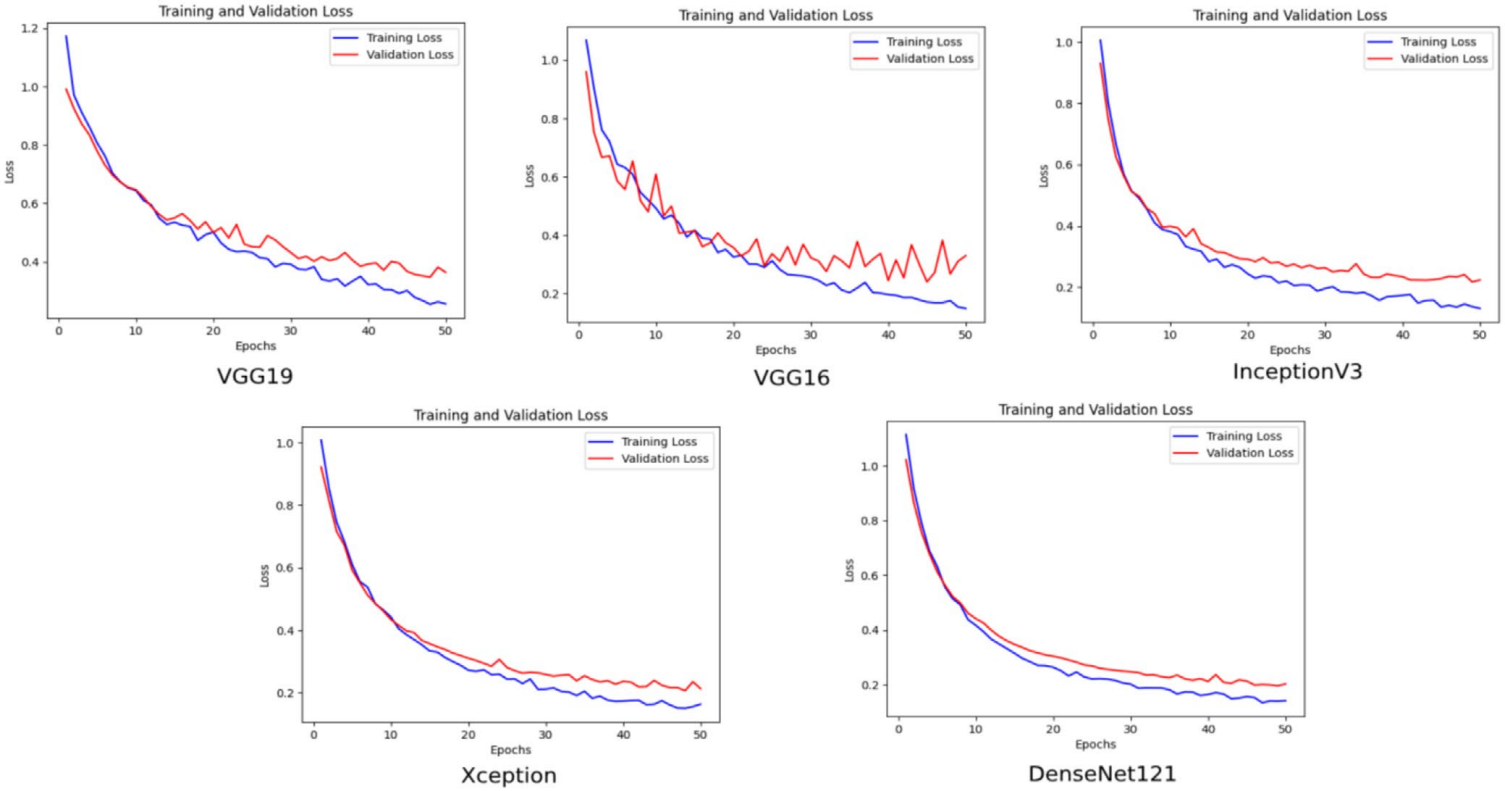
Comparison of training and validation loss graphs of pre‐trained networks.

The change in training and validation loss values over time for deep learning models (VGG19, VGG16, InceptionV3, Xception, and DenseNet121) is presented in Figure 8. The graphs show the learning dynamics of the models during their training processes. In all models, training loss and validation loss values are high at the beginning and tend to decrease as training progresses. This shows that the models are learning from the data.

DenseNet121 and Xception models show the most stable decreasing trend in validation loss values, revealing that they have better generalization capacity.

**FIGURE 9:**
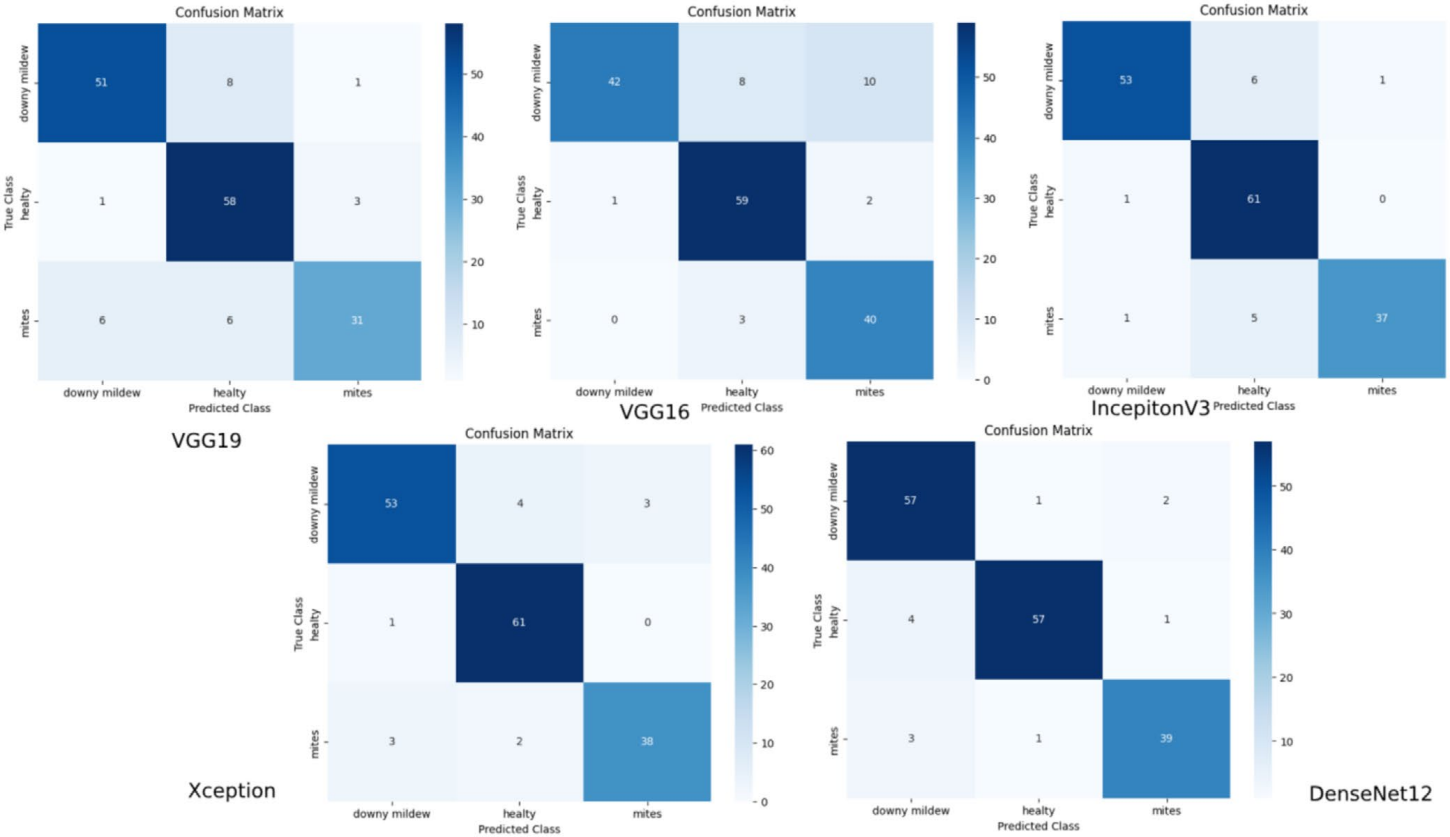
Comparison of confusion matrices for pre‐trained networks.

The complexity matrices in Figure 9 show the classification performance of different deep learning models in detail.

When we examine the complexity matrix for the VGG19 model, a high correct classification rate was observed, especially in the “downy mildew” and “healthy” classes. However, the errors were higher in the “mites” class; some samples were misclassified as “downy mildew” and “healthy.” This shows that the model has difficulty distinguishing the “mites” class. When we examine the VGG16 model, similarly, although it exhibited a strong performance in the “healthy” class, the error rate was higher in the “downy mildew” and “mites” classes. The false positive rate in the “downy mildew” class is particularly striking. When we look at the complexity matrix for the InceptionV3 model, it showed a relatively balanced performance for all classes. While almost perfect accuracy was achieved in the “healthy” class, small errors were observed in the “mites” and “downy mildew” classes. When we look at the complexity matrix for Xception, it provided better accuracy in the “mites” class compared to the other models. However, there are small errors in the “downy mildew” and “healthy” classes. It can be said that it has a good performance in general. DenseNet121 showed the most balanced performance in all classes. Especially in the “downy mildew” and “healthy” classes, the correct classification rate is quite high. It also made fewer errors than other models in the “mites” class.

**FIGURE 10:**
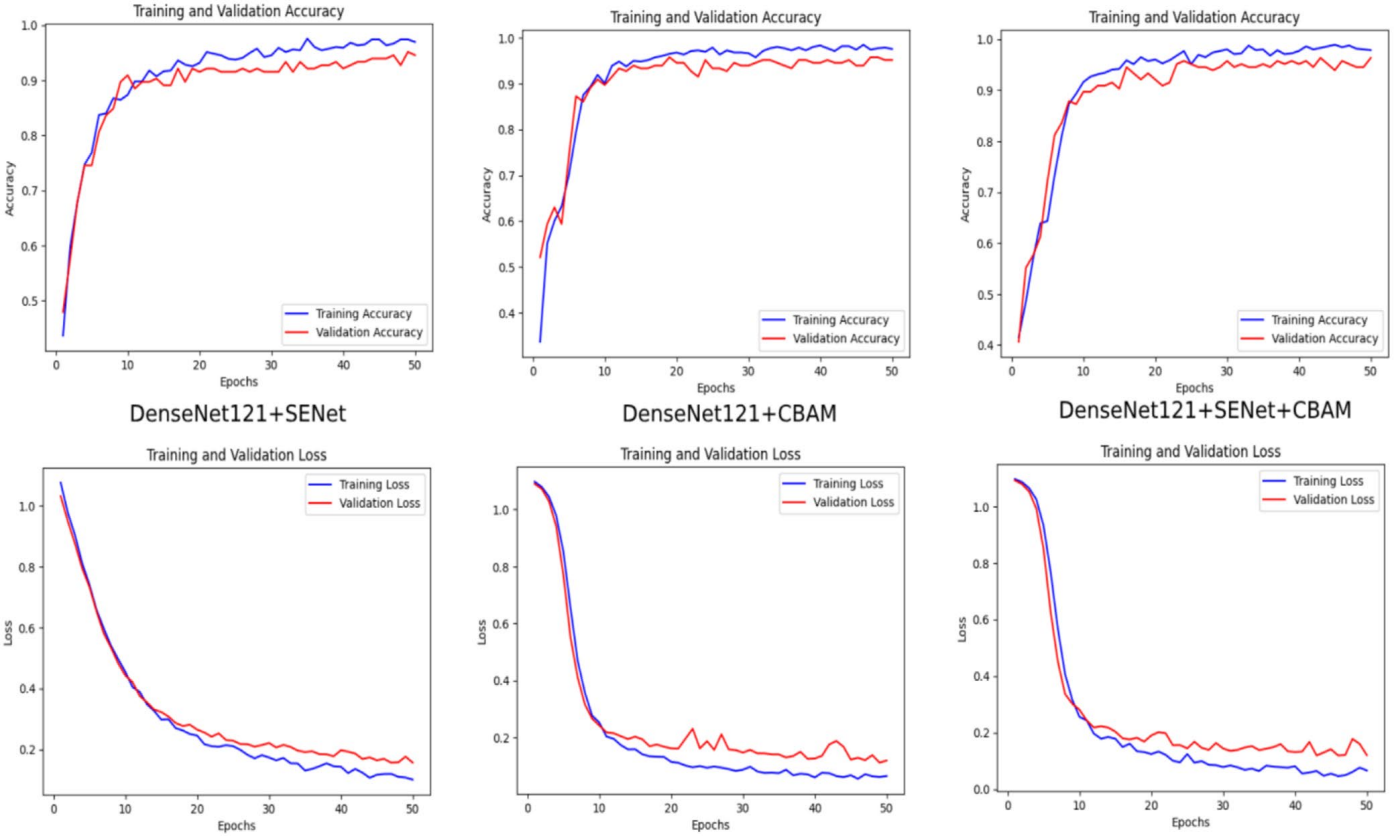
Training and validation analysis of DenseNet121 + SENet and CBAM.

The training and validation processes of the DenseNet121 + SENet, DenseNet121 + CBAM, and DenseNet121 + SENet + CBAM models are given in Figure 10. Training and validation accuracy and training and validation loss are given for each model.

The upper graphs show the change in accuracy, and the lower graphs show the change in loss. In DenseNet121 + SENet, training and validation accuracy increased rapidly at first and stabilized around epoch 20–30. Training accuracy was slightly higher, but validation accuracy was very well matched. Training loss and validation loss decreased rapidly, and although validation loss showed some fluctuations, it generally remained at a low level. For DenseNet121 + CBAM, training accuracy and validation accuracy increased rapidly again and stabilized after epoch 30. Validation accuracy is quite consistent with training accuracy. Training and validation loss generally reached low levels. However, it is seen that there are small fluctuations in validation loss as in other models. For DenseNet121 + SENet+CBAM, training and validation accuracy follow a similar course with other models, but validation accuracy is observed to be more stable. Validation accuracy is one of the most consistent in this model. Training loss decreased steadily, and validation loss is generally low. However, there is less fluctuation towards the end of the epochs, indicating that the model exhibits better generalization performance. In the analysis, DenseNet121 + SENet+CBAM was the most successful generalization model. The close training and validation accuracies showed that all models did not overfit. The combination of CBAM with SENet gave the most stable results in validation accuracy and loss. This combination better captures complex features.

The variations observed in the comparative analysis plots can be explained by several factors. The first set of plots (Figure 7) shows the training and validation accuracy curves of different deep learning architectures (VGG19, VGG16, InceptionV3, Xception, DenseNet121). The structural differences between the architectures cause variations in learning rate and convergence behavior. The third set of plots (Figure 10) shows that the variations in the accuracy curves decrease, especially with the inclusion of attention mechanisms (SENet, CBAM and their hybrid versions). This is due to the fact that attention mechanisms improve the ability of the model to focus on important features.

**FIGURE 11:**
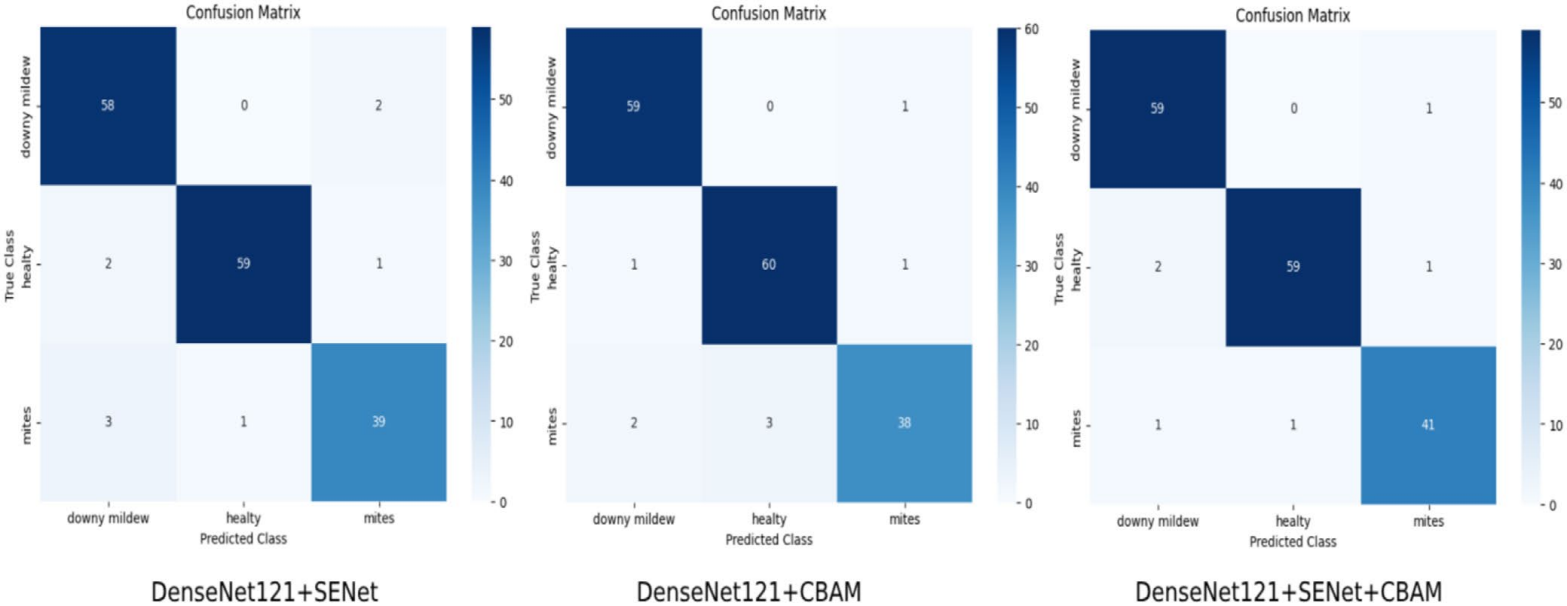
Comparison of confusion matrices for DenseNet121 + SENet, DenSeNet121 + CBAM, DenseNet121 + SENet + CBAM.

In Figure 11, the confusion matrices created for DenseNet121 + SENet, DenseNet121 + CBAM, and DenseNet121 + SENet + CBAM are given. These matrices show the classification performances of the models. Each cell represents the number of class examples that the model predicted correctly or incorrectly.

Looking at Figure 11, the classification performance of the models is analyzed for three classes (downy mildew, healthy, and mites). Looking at the confusion matrix for DenseNet121 + SENet, for the downy mildew class, there are 58 correct predictions and 2 incorrect predictions (classified as mites). For the healthy class, there are 59 correct predictions, and 2 incorrect predictions (classified as 1 mites, 1 downy mildew). For the mites class, there are 39 correct predictions and 4 incorrect predictions (classified as 3 downy mildew, 1 healthy). When we look at the overall performance of the DenseNet121 + SENet model, the performance of the model is generally good, but there are more incorrect predictions in the mites class compared to other classes. Looking at Figure 11 for DenseNet121 + CBAM, for the downy mildew class, there are 59 correct predictions and 1 incorrect prediction (classified as mites). For the healthy class, there are 60 correct predictions and 1 incorrect prediction (classified as mites). For the mites class, there are 38 correct predictions and 5 incorrect predictions (2 classified as downy mildew, 3 as healthy). When we look at the overall performance of the DenseNet121 + CBAM model, it is seen that it is more successful in the downy mildew and healthy classes. However, there are still some incorrect classifications in the mites class. When Figure 11 is examined for DenseNet121 + SENet + CBAM, there are 59 correct predictions and 1 incorrect prediction (classified as mites) for the downy mildew class. There are 59 correct predictions, and 2 incorrect predictions (2 classified as downy mildew) for the healthy class. There are 41 correct predictions and 2 incorrect predictions (1 classified as downy mildew, 1 as healthy) for the mites class. In terms of overall performance, it is the model that made the least incorrect predictions in the mites class. When we look at the overall performance of DenseNet121 + SENet + CBAM, it exhibited a more balanced performance compared to other models in all classes.

**FIGURE 12:**
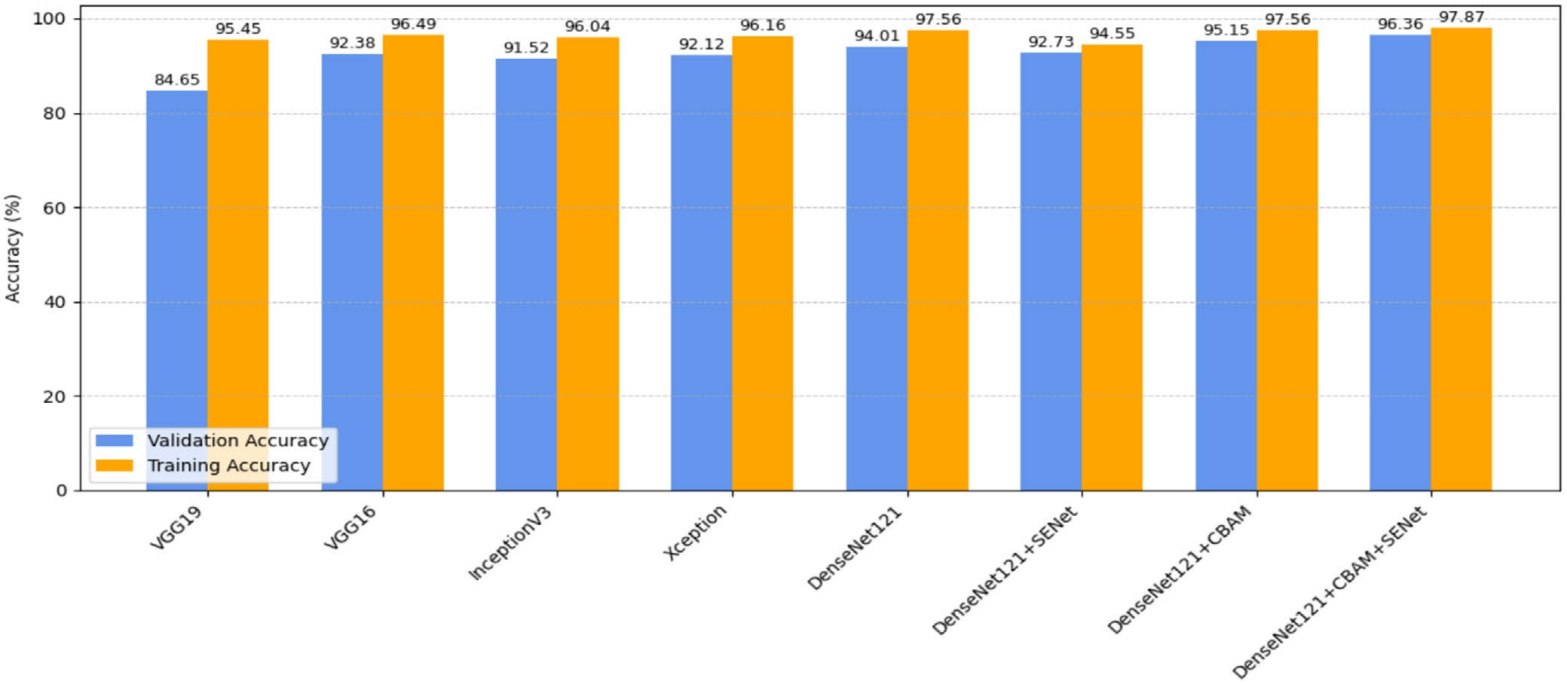
Comparison of validation and training accuracy across different models.

The graphs regarding the training and validation processes of the pre‐trained models used in the study and the proposed models are presented in Figure 12.

As a result, the findings show that methods that take into account attention mechanisms and channel relationships provide a significant advantage in grape leaf disease classification. In particular, CBAM and SENet components built on the DenseNet121 architecture provided superior accuracy in both training and validation stages by providing better feature extraction from visual data. These findings can serve as a reference for future studies and guide model optimization. In addition, the performance of the supported models (CBAM, SENet) has yielded results at a level that is competitive with many studies in the literature. Our DenseNet121 + CBAM + SENet model has shown a performance close to or superior to other studies in the literature with 96.36% validation accuracy. The results of the performance improvement are given in Table 4.

**TABLE 4:** Methodology and performance improvement in grapevine leaf disease detection.

Authors	Methods	Parameters	Improved accuracy/performance
Proposed method	DenseNet121 + CBAM + SENet	Epoch: 50, LR: 0.0001, Optimizer: Adam, Batch size: 32, Activation Func: RELU	96.36% (92.73 ➔ +3.66)

The proposed method integrates DenseNet121, CBAM (Convolutional Block Attention Module), and SENet (Squeeze‐and‐Excitation Networks) for the classification of grapevine leaf diseases. The model was trained for 50 epochs with a learning rate of 0.0001, using the Adam optimizer, a batch size of 32, and the ReLU activation function. This configuration resulted in an improvement in accuracy, achieving 92.73% accuracy, which represents an enhancement of 3.66% compared to previous results. The use of attention mechanisms such as CBAM and SENet likely contributed to better feature selection and improved model performance, highlighting the effectiveness of combining these advanced techniques.

Although the integration of the CBAM and Squeeze‐and‐Excitation (SE) modules into the baseline model leads to a slight increase in computational time during both training and inference, this overhead remains minimal and within acceptable limits for most practical use cases. The computational complexity increase introduced by the proposed hybrid attention mechanism is entirely reasonable and justifiable considering the performance improvement and classification accuracy gain it provides. Moreover, with modern GPU hardware, this overhead does not pose a significant obstacle in practical applications.

## 5 Conclusions

Diseases seen on grape leaves can negatively affect the development of the plant and damage many products obtained from the grapevine. Especially diseases such as grape leaf blister mite and downy mildew can seriously limit the use of grape leaves in traditional cuisines. This situation causes individuals engaged in viticulture to experience serious economic losses. In addition, these diseases negatively affect plant development and seriously damage grape fruit. Farmers experience financial losses both from the products to be obtained from the plant leaves and from the grape fruit. In this study, a basic classification process was performed using pre‐trained neural networks for the classification of grape leaf diseases (three classes: grape leaf blister mite and downy mildew and healthy). In order to increase the model performance, Convolutional Block Attention Module (CBAM) and squeeze‐and‐excitation networks (SE) modules were integrated. This integration increased the classification accuracy from 92.73% to 96.36%, demonstrating the effectiveness of attention mechanisms. This study provides an effective solution for early and accurate detection of leaf diseases, which is an important problem encountered in agricultural production. The approach that integrates the attention mechanisms of CBAM and SE modules has significantly increased the classification accuracy and is a reference for similar studies carried out in this field. The obtained results strengthen the potential of using artificial intelligence‐based solutions in large‐scale agricultural applications. The findings of the study allow for both the improvement of deep learning models for future research and the development of more sensitive and economical applications in the agricultural sector. The integrated use of CBAM and squeeze‐and‐excitation (SE) networks provides remarkable advantages in the diagnosis of grapevine leaf diseases. In particular, the ability of attention mechanisms to emphasize important features has increased the diagnostic accuracy. The study provides an effective solution for early diagnosis of various grapevine leaf diseases, while emphasizing the importance of increasing generalization capacity with rich and diversified data sets. Our study was collected from a region where grapevine leaves have an important economic value. This study was conducted for the two diseases that are most common in this region and directly damage grapevine leaves. In future studies, it can be applied to other diseases seen in grape leaves. In addition, other deep learning models can be used to increase the classification performance. In future studies, classification performance can be improved with various hyperparameter optimization techniques as well as different deep learning models. Hyperparameter optimization can be performed using meta‐heuristic approaches such as grid search and random search, genetic algorithms, and particle swarm optimization. The developed methodology has the potential to be integrated into real‐time grapevine leaf disease detection systems in future studies. Implementation of such real‐time applications in field conditions can increase the effectiveness of agricultural interventions by enabling early detection of disease spread and thus contribute to sustainable viticulture practices. For this study, we used our own dataset, and the number of images in the dataset can be increased in future studies. Working with a larger dataset can also increase the classification success. The dataset used in the study was specially created for this research. The fact that the data was collected from a specific region and a limited number of grapevine species constitutes one of the potential limitations that should be considered within the scope of the study. In addition, imbalances between the classes in the dataset are considered as another obstacle to improving the classification performance to a higher level. In addition, the exposure of the images obtained in the real vineyard environment to different lighting conditions stands out as another limitation that may affect the overall success of the model. The limited number of images in the dataset is another issue that should be considered in this context. Various strategies can be applied to overcome the mentioned limitations. In order to increase the diversity of the dataset, more comprehensive data collection studies can be carried out from different regions and different grapevine species. In this way, the generalizability of the model to different environmental conditions and grapevine species can be increased. Data augmentation techniques can be used to reduce class imbalances in the dataset; for example, image augmentation methods can be applied for underrepresented classes. In order to minimize the effect of variable light conditions in the real vineyard environment on model performance, it is advisable to use a specially designed image capture platform in addition to image preprocessing processes.

## Author Contributions

**Yavuz Unal:** conceptualization (lead), data curation (lead), formal analysis (lead), investigation (lead), methodology (lead), project administration (lead), resources (lead), software (lead), supervision (lead), validation (lead), visualization (lead), writing – original draft (lead), writing – review and editing (lead).

## Conflicts of Interest

The author declares no conflicts of interest.

## Data Availability

Data supporting the findings of this study are available from the corresponding author upon reasonable request.
